# Patchy Charge Distribution Affects the pH in Protein Solutions during Dialysis

**DOI:** 10.1021/acs.langmuir.4c04942

**Published:** 2025-02-18

**Authors:** Sebastian P. Pineda, Pablo M. Blanco, Roman Staňo, Peter Košovan

**Affiliations:** † Department of Physical and Macromolecular Chemistry, Faculty of Science, Charles University, Hlavova 8, 128 40 Prague 2, Czech Republic; ‡ Department of Physics, NTNU - Norwegian University of Science and Technology, NO-7491 Trondheim, Norway; § Faculty of Physics, University of Vienna, Boltzmanngasse 5, 1090 Vienna, Austria; ∥ Vienna Doctoral School in Physics, University of Vienna, Boltzmanngasse 5, 1090 Vienna, Austrias

## Abstract

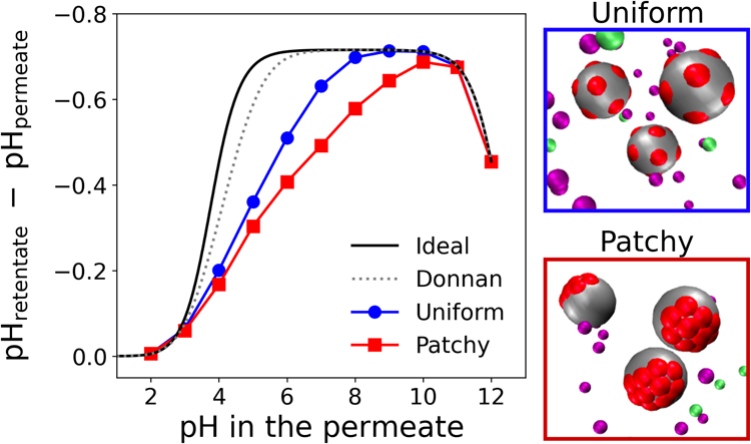

When using dialysis ultra- or diafiltration to purify protein solutions, a dialysis buffer in the permeate is employed to set the pH in the protein solution. Failure to achieve the target pH may cause undesired precipitation of the valuable product. However, the pH in the permeate differs from that in the retentate, which contains the proteins. Experimental optimization of the process conditions is time-consuming and expensive, while accurate theoretical predictions still pose a major challenge. Current models of dialysis account for the Donnan equilibrium, acid–base properties, and ion–protein interactions, but they neglect the patchy distribution of ionizable groups on the proteins and its impact on the solution properties. Here, we present a simple computational model of a colloidal particle with weakly acidic sites on the surface, organized in patches. This minimalistic model allows systematic variation of the relevant parameters, while simultaneously demonstrating the essential physics governing the acid–base equilibria in protein solutions. Using molecular simulations in the Grand-Reaction ensemble, we demonstrate that interactions between ionizable sites significantly affect the nanoparticle charge and thereby contribute to pH difference between the permeate and retentate. We show that the significance of this contribution increases if the ionizable sites are located on a smaller patch. Protein solutions are governed by the same physics as our simple model. In this context, our results show that models which aim to quantitatively predict the pH in protein solutions during dialysis need to account for the patchy distribution of ionizable sites on the protein surface.

## 1 Introduction

Dialysis is a ubiquitous unit operation used in various branches of chemical industry. Along with ultra- and diafiltration (UF/DF), it is used in the pharmaceutical sector for purification of protein solutions and increasing their concentration.^1^ This unit operation uses two spatial compartments: the retentate, which contains the protein solution, and the permeate, which contains various excipients–small molecules and ions. These two compartments are separated by a size-selective semipermeable membrane that enables the exchange of small molecules and ions between the retentate and permeate, while preventing the exchange of proteins and other macromolecules. A dialysis buffer is used to maintain a certain pH in order to ensure the desired properties of the protein solution.^2^ Failure to maintain the desired pH in the retentate may cause aggregation or precipitation of the proteins, which could result in the loss of the final product. However, pH in the dialyzed protein solution at equilibrium usually differs from that in the buffer, and the same is true for the concentrations of excipients which can be exchanged between the two compartments.^3−5^

The pH difference between retentate and permeate is primarily determined by the *Donnan equilibrium*,^6,7^ that depends on the charge on the proteins and other macromolecules or colloidal particles which cannot be exchanged between the permeate and retentate. Simultaneously the charge on the proteins is regulated by pH in the retentate. The complicated feedback loop between the Donnan equilibrium, pH in the retentate and protein charge causes that controlling the excipient concentrations and pH in the retentate is a major challenge. Empirical trial-and-error process optimization becomes very expensive, especially if it concerns purification of a high-value product, such as monoclonal antibodies. Therefore, various modeling approaches have been developed with the intention to accurately predict the pH and excipient concentrations in dialysis and UF/DF unit operations.^4−13^ The goal of the modeling is to predict the properties of the retentate at a given composition of the permeate. Current state-of-the-art modeling approaches can quantitatively predict experimentally observed pH and excipient concentrations in dialyzed protein solutions under certain conditions, while they may fail if a different protein or a different buffer is used.^5,11,12^ This discrepancy indicates that some important features of the protein solutions have been neglected in these models. However, it is not *a priori* clear which are the key missing features.

### 1.1 Donnan Equilibrium

The theoretical modeling of dialysis starts from the Donnan equilibrium, sometimes termed Gibbs-Donnan to acknowledge that actually Gibbs described it first.^14^ The theoretical predictions typically focus on predicting how the excipient concentrations vary in time, as the process conditions are being varied. Nevertheless, they assume a quasi-equilibrium state, i.e. that the Donnan equilibrium is established much faster than the time scale on which the process conditions are varied. The permeate is usually present in excessive amount as compared to the retentate. Therefore, the models treat the permeate as an infinitely big reservoir of a constant composition. The Donnan equilibrium is based on the ideal gas approximation, combined with the electroneutrality approximation. To satisfy electroneutrality, charge on the macromolecules in the retentate has to be compensated by an equivalent amount of oppositely charged counterions, causing that the counterions are distributed asymmetrically between the retentate and permeate. This asymmetric distribution can be quantified in terms of distribution ratio
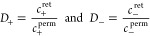
1where *perm* refers to the permeate or the dialysis buffer, *ret* refers to the retentate or the protein solution, “+” refers to monovalent cations and “–” refers to monovalent anions. The Donnan equilibrium is established when the electrochemical potentials of all ion pairs in the retentate and permeate are equal.^3,15^ Within the ideal gas approximation, the distribution ratios of cations and anions are determined by the Donnan potential, ψ_Don_
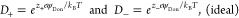
2where *z* is the ion valency, *e* is the elementary charge, *k*_B_ is the Boltzmann constant and *T* is temperature. Here and below, the text ”ideal” next to the equation refers to the fact that it is exact only within the ideal gas approximation. Equation 2 implies that the distribution coefficients of all monovalent cations are equal and inversely propotional to those of monovalent anions^16^
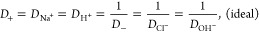
3Importantly, equivalence of distribution ratios for monovalent ions in eq 3 applies not only to ideal systems but also to interacting ones, as long as various monovalent ions are represented using the same interaction potentials. In particular, *D*_H^+^_, determines the difference in pH between the permeate and retentate
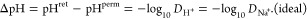
4Notably, eq 4 is exact for ideal systems but it also works as a good approximation for nonideal systems because the activity coefficients in the retentate and permeate tend to have similar values and therefore cancel each other. As long as the distribution ratios of monovalent ions are related via eq 3, the value of ΔpH can be quantified using the distribution ratio of any monovalent cation.^15^

Without loss of generality, we will further assume that proteins in the retentate are negatively charged and we will focus on the distribution of cations, noting that the same arguments apply to positively charged proteins, just that the roles of cations and anions are swapped in all equations. If the concentration of nonexchangeable anionic charges A^–^ in the retentate is given by *c*_A^–^_, then the distribution ratio can be expressed as^16^
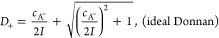
5where
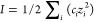
6is the ionic strength of the permeate (dialysis buffer). The summation index *i* in eq 6 runs over all small ions present in the system. Equation 5 has two limiting cases:
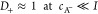
7
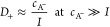
8Typical experimental conditions in dialysis of protein solutions feature highly concentrated protein solutions and moderate ionic strengths of the buffer. In such case, eq 8 is the relevant limit.

### 1.2 Acid–Base Equilibrium Coupled to the Donnan Equilibrium

**Figure 1:**
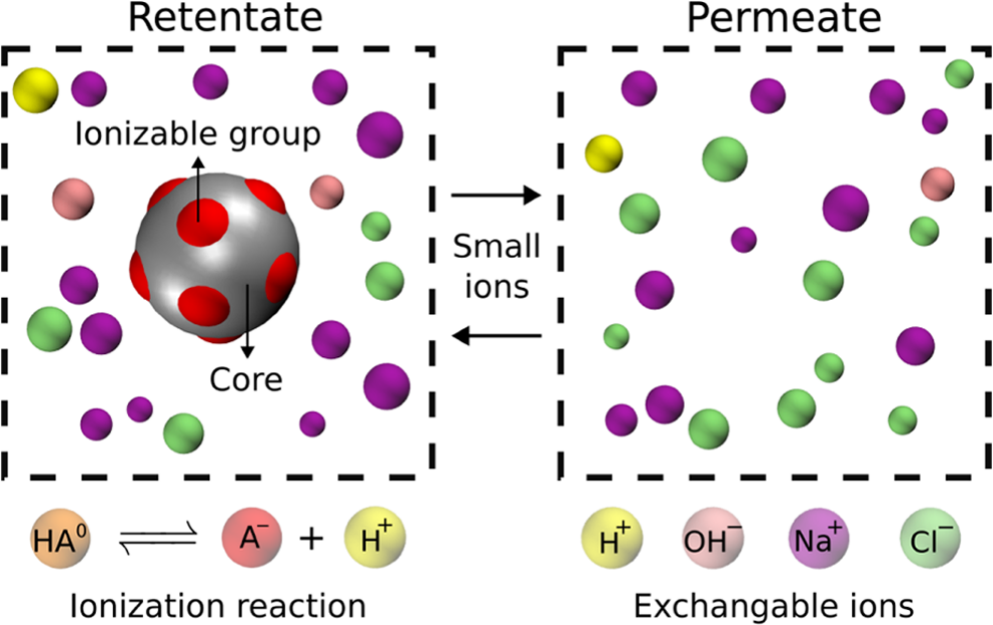
Schematic illustration of the studied system: a solution of nanoparticles with acidic ionizable groups and small ions (H^+^, OH^–^, Na^+^ and Cl^–^) on the left (retentate), coupled to permeate containing only small ions on the right.

The charge on the proteins is determined by the acid–base equilibrium of their ionizable residues. Again, without loss of generality, we limit the discussion to acidic residues, as schematically illustrated in Figure 1. Note that an analogous description applies to basic residues and that the net charge on the protein is given as a sum of the charges of all residues.

The dissociation of a weak acid group HA is described by the following equation
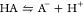
9and quantified by the degree of ionization, α, defined as
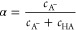
10where subscripts refer to ionized and nonionized forms of the acid, defined by eq 9. We emphasize that *c*_A^–^_ and *c*_HA_ in eq 10 are the equilibrium concentrations of the A^–^ and HA groups on the proteins in the retentate. The ionization degree is coupled to pH via the Henderson–Hasselbalch equation (HH)
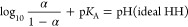
11Note that, in some textbooks, the name Henderson–Hasselbalch equation is used for a similar-looking equation, which is used to calculate the pH of buffer solutions from the analytical concentrations of weak acid and a strong base.^17^ In contrast, other textbooks use the same name in a more general context, referring to equations analogous to our eq 11, which use the logarithm of equilibrium concentrations of an acid and its conjugate base.^18^ An important difference is that the form using the equilibrium concentrations of conjugate acid/base pair is exact, whereas the one using analytical concentrations is just an approximation with a limited validity.^19,20^

When considering the acid–base equilibrium in a membrane-separated system, it is important to recognize that eq 11 refers to pH in the retentate. To express the ionization degree as a function of pH in the permeate, it is necessary to account for ΔpH due to the Donnan partitioning, via eq 4
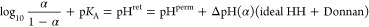
12where we have explicitly indicated that ΔpH depends on α. This coupling closes the feedback loop between the Donnan equilibrium and charge regulation on the proteins. Interactions in real systems cause deviations from the ideal equations given above. Accounting for nonideality of both retentate and permeate has been the main challenge of all modern theories.

### 1.3 Acid–Base Equilibrium in Protein Dialysis

Currently used models account for protein-ion interactions and for the excluded volume of the proteins on the mean-field level, representing the proteins as spherically symmetric particles with charge homogeneously distributed on the surface.^4−6^ By solving the spherically symmetric Poisson–Boltzmann equation, they determine the concentration profiles of small ions around the protein in the center of a spherical Wigner-Seitz cell, which as a whole must be electroneutral. The volume of the Wigner-Seitz cell is determined by the protein concentration, assuming a fixed protein charge.^6^ The sphere representing the protein is impenetrable for ions to account for the protein volume while the contribution of excipients can be included via their partial molar volumes.^7^ This model could effectively reproduce the deviations in excipient concentration and pH at certain conditions, but failed when applied to a different type of monoclonal antibodies at higher concentrations.^7^ Other theories include the variation of protein charge as a function of pH in the retentate using a simple mass balance model, accounting for protein-ion binding but not considering the ion distribution around the protein, as in the previous models. Such approaches yield accurate predictions in histidine buffer but not in succinate buffer.^4^ Another model accounting for the variation of protein charge as a function of pH was introduced by Baek et al.^8^ In their model, the protein charge was determined from independent electrophoretic mobility measurements, whereas concentrations of different ionizable species in the buffer were calculated from the Henderson–Hasselbalch equation, including the correction for activity coefficients.^8^ The model introduced by Jabra et al.^9^ incorporated the effects of both pH and ionic strength on the protein charge into a mass balance model to describe the pH and excipient profiles during dialysis. In this study, the net charge on the protein was determined as the sum of the fractional charges on individual residues. It employed the Henderson–Hasselbalch equation, accounting for the local electrostatic potential at the protein surface, which was estimated from an approximate analytical expression. This theoretical approach successfully reproduced the deviations in excipients and pH of the monoclonal antibody solutions used in their study. A similar approach was used in the mean-field model proposed by Boubeta et al.,^21^ where the charges of the ionizable sites were fixed at fractional values determined by the average ionization degree, computed from the Poisson–Boltzmann equation. Nevertheless, this approximation is prone to failure in systems with significant charge correlations and fluctuations, as has been observed in models containing both acidic and basic ionizable sites.^22−25^ For example, Yuan et al.^22^ showed that the self-organization behavior in a suspension of overall-neutral zwitterionic Janus nanoparticles with fluctuating charges fundamentally differs from the commonly employed constant-charge approximation. In this study, charge regulation triggers nanoparticle clustering in systems with inhomogeneous and fluctuating charged patches, while this phenomenon is absent when fixed charges are used. These structural changes are accompanied by changes in the degree of ionization of both acidic and basic sites and, consequently, we can expect an effect of charge fluctuations on the ΔpH in the context of dialysis.

Finally, Briskot et al.^5^ combined all previous components in a complex model which employed Donnan equilibrium, pH-dependent charge of both protein and excipients, distribution of ions and surface potential on the protein calculated from the Poisson–Boltzmann equation, and activity coefficients of small ions. This model accurately predicted pH and excipient concentrations of one type of monoclonal antibodies in acetate, succinate and histidine buffers. However, for another type of antibodies, it slightly deviated in succinate buffer at pH > 5.6 and significantly deviated in histidine buffer at pH > 6. These observations suggest that, despite numerous corrections introduced in the models, some important features are still missing.

None of the theoretical models of protein dialysis known to us has explicitly accounted for inhomogeneous charge distribution on the protein surface, although there are indications in the literature that it might have a significant effect. For example, the impact of patchy charge distribution on the net charge of the protein has been discussed in the context of charge regulation, *i.e*. change of the ionization state at a given pH in response to changes in the local environment. Charge regulation causes that interactions between individual ionizable groups on the protein surface affect the protein net charge.^26−28^ These charge-patch interactions have been found to affect the phase separation of monoclonal antibodies^29^ and supercharged proteins.^30,31^

### 1.4 Charge Regulation

Models accounting for charge regulation effects in proteins are commonly based on solving the Poisson–Boltzmann equation coupled to Henderson–Hasselbalch equation for each ionizable site. The latter accounts for the local electrostatic potential at a given site, computed from the Poisson–Boltzmann equation in three dimensions, which is more complex than the one-dimensional spherical approximation employed in the models of dialysis and UF/DF.^32−34^ In this approach, the positions of ionizable sites are obtained from proteins structures stored in the PDB database. Such models could very accurately predict the p*K*_A_ shifts in various proteins.^27^

Apart from protein research, the effects of charge patchiness have been studied in the context of charged colloids, polyelectrolytes and polyelectrolyte-protein interactions. Poisson–Boltzmann theories have been developed to describe charge regulation in colloids, based on spherical symmetry approximation, akin to the models used for predicting dialysis.^35,36^ The charge regulation effects in colloids and polyelectrolytes typically yield a lower net charge than predicted by Henderson–Hasselbalch equation. This effect has been known in the field of polyelectrolytes since the early 1950s^37,38^ and since then it has been observed in various experimental studies, e.g., refs. (39,40) The decreased net charge is a direct consequence of repulsion between like-charged ionizable sites on synthetic polyelectrolytes, which increases the free energy cost of extra ionization. The key parameter that determines this extra free energy cost is the distance between the ionizable sites.^41−43^ This effect has been termed *Polyelectrolyte effect*, to distinguish it from the *Donnan effect*, which has similar consequences but a different physical origin.^16^

If both positive and negative charges are involved, then charge regulation is controlled by the delicate balance between the repulsion among like charges and attraction among opposite charges. In such cases, charge regulation may lead to attraction and aggregation,^22,44^ similar as has been observed for patchy colloids with a fixed charge.^45−49^ Thanks to charge–charge correlations, polyelectrolytes are attracted to oppositely charged patches on proteins and peptides, while avoiding the like-charged patches.^50,51^ The attraction to charge patches explained the counterintuitive adsorption of proteins to polyelectrolytes on the ”wrong” side of the isoelectric point, *i.e*. at pH values when net charge on the protein has the same sign as the polyelectrolyte.^52,53^ Later, it has been shown that also charge regulation can trigger attraction under specific conditions, when it would not be expected based on the ideal Henderson–Hasselbalch model.^25,54,55^ The latter findings have been obtained using molecular simulation models which account for conformational changes of the polymers, site–site and ion–ion correlations beyond the mean-field approximation used in the Poisson–Boltzmann equation. Importantly, the above account of literature on the combined effect of charge patchiness and charge regulation is far from being exhaustive. Nevertheless, we believe that it sufficiently illustrates the important role of charge patchiness in protein–protein, protein-colloid and protein-polyelectrolyte interactions.

The performance of all previously discussed models of dialysis or UF/DF has been assessed based on macroscopic parameters, obtained from the experiments. Such an assessment may be tricky because various features result in qualitatively similar trends, while the quantitative results depend on the involved approximations. To get a deeper insight into the importance of specific model features under various conditions, we present molecular simulations using a simple microscopic model of a colloidal particle with patchy charge distribution, which allows us to go beyond the approximations used in the macroscopic models. Using the Grand-Reaction method,^16^ we simulate a solution of these particles in equilibrium with a reservoir of small ions, emulating a protein solution in the retentate, dialyzed against a buffer solution in the permeate. Our model is constructed as a proof of concept to demonstrate the impact of charge patchiness, electrostatic interactions and steric effects on dialysis, ultra/diafiltration and other membrane-based separation processes.

## 2 Materials and Methods

### 2.1 Simulation Model

Our model consists of a minimalistic representation of a protein solution (retentate) separated from a buffer solution (permeate) by a semipermeable membrane. The membrane is not simulated explicitly in our simulation setup. Instead, we represent bulk sections of both retentate and permeate, assuming that they have reached equilibrium, as schematically illustrated in Figure 1. The retentate contains nanoparticles with ionizable acidic sites, as a minimalistic coarse-grained representation of a protein, accounting for its acid–base properties, electrostatic interactions and excluded volume. In addition to these nanoparticles, small monovalent ions are present in the simulated system, representing excipients in the dialysate and components of the dialysis buffer. The small ions can be exchanged between the retentate and permeate until thermodynamic equilibrium is reached. We set the pH and concentrations of small ions in the permeate as inputs of the simulation, and obtain the ionization degree or nanoparticles, pH and ion concentrations in the retentate as outputs.

The small ions (Na^+^, Cl^–^, H^+^ and OH^–^) are represented as spherical particles with elementary charge *z* = ± 1e and diameter *d*_ion_ = 0.355 nm. The diameter of ions was chosen such that their chemical potential reasonably approximates the chemical potential of aqueous solutions of NaCl almost up to the solubility limit.^16,56^ Each nanoparticle is composed of a repulsive core and acidic ionizable groups on the surface. The core and ionizable sites are represented as a single rigid object which can rotate but cannot change its shape or positions of the ionizable sites. Unless specified otherwise, the nanoparticles have a core with effective diameter *d*_np_ = 1.42 nm and with 10 weakly acidic groups with p*K*_A_ = 4.0 on the surface. The core diameter *d*_np_ = 4*d*_ion_ has been chosen to ensure that the nanoparticles are significantly bigger than monovalent ions, yet not too big, so that the computational costs of the simulations remain reasonable.

To verify the importance of charge fluctuations, we also performed a set of simulations using nanoparticles containing fully ionized groups with a specific fractional charge. This fractional charge was fixed to the average ionization degree, obtained from the same simulations with charge fluctuations.

The simulation box, representing the retentate, was coupled to a virtual reservoir of ions, representing the permeate. The exchange of ions between the simulation box and the reservoir was modeled via the Grand-Reaction Monte Carlo method,^16^ as detailed in Section 2.2. This method ensures that all ion pairs in the retentate and permeate have the same chemical potentials, representing a dialysis process at equilibrium stage. Properties of the reservoir are defined by the chemical potentials of its constituents, which are the input parameters of the simulation. Composition of the reservoir with the given parameters is determined from an independent auxiliary simulation.

#### 2.1.1 Distribution of Ionizable Sites

The patchy nanoparticles contain a single patch that houses all ionizable sites. To achieve an approximately uniform distribution of sites on a given patch, we used a numerical procedure to place individual sites, minimizing the sum of pair distances between them. The fraction of surface occupied by the patch is given by (1 – θ), where θ is the degree of patchiness. Thus, at θ = 0, the ionizable sites are approximately uniformly distributed across the whole nanoparticle, whereas at θ = 0.90 the sites are uniformly distributed only on 10% of the nanoparticle surface, as illustrated in Figure 5A. The resulting average distances between the sites on the nanoparticles, *d*_q_, and their ratios to the Bjerrum length, *d*_q_/λ_B_, are listed in Table 1.

**Table 1:** Properties of the Distributions of Ionizable Sites on the Nanoparticles: Degree of Patchiness, θ, Average Distance between the Ionizable Sites, *d*_q_, and Its Ratio to the Bjerrum Length, *d*_q_/λ_B_

degree of patchiness, θ	average distance between sites, *d*_q_ [nm]	*d*_q_/λ_B_
0.00	0.54 ± 0.02	0.76 ± 0.02
0.50	0.41 ± 0.01	0.58 ± 0.02
0.75	0.30 ± 0.01	0.42 ± 0.01
0.90	0.21 ± 0.01	0.30 ± 0.02

#### 2.1.2 System Size and Nanoparticle Volume Fraction

The size of the simulation box was chosen such that it should contain approximately *N*_salt_ = 250 salt ion pairs Na^+^Cl^–^, using the formula *V*_box_ = *N*_salt_/(*N*_A_*c*_salt_) where *c*_salt_ is the salt concentration in the permeate. Then, the number of nanoparticles was set to an integer value, *N*_np_, resulting in the nanoparticle volume fraction, defined as
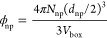
13At any simulated combination of *c*_salt_ and ϕ_np_, we always had *N*_np_ ≥ 10 to suppress possible finite-size effects.^57^

#### 2.1.3 Interaction Potentials

We used an augmented version of the Weeks–Chandler–Andersen (WCA) potential to account for the steric repulsion between particles with different sizes
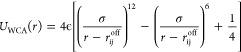
14for *r*_*ij*_^off^ < *r* < *r*_cut_ + *r*_*ij*_^off^, where *r* is the distance between the interacting particles, *r*_cut_ is the cutoff distance, beyond which the interaction vanishes. The parameter σ determines the effective range of repulsion and *r*_ij_^off^ is the offset distance, below which the interaction potential is infinite. The value of σ was the same for all interactions whereas the value of *r*^off^ was used to adjust the interaction range, based on the desired effective particle size. The distance at contact between two particles *i* and *j* is given by *r*_*ij*_^off^ + σ = (*d*_*i*_ + *d*_*j*_) /2 where *d*_*i*_ and *d*_*j*_ are their effective diameters. For ion–ion interactions, we used *r*_ij_^off^ = 0 and σ = *d*_ion_ = 0.355 nm. For nanoparticle-ion interactions, we used *r*_np–ion_^off^ = (*d*_np_ + *d*_ion_)/2 – σ = 0.5325 nm, and for nanoparticle–nanoparticle interactions we used *r*_np–np_^off^ = *d*_np_ – σ = 1.065 nm. By using *r*_cut_ = 2^1/6^ σ, we made the potential purely repulsive. Then, the prefactor ϵ = 1*k*_B_*T* was chosen arbitrarily to determine the energy scale.

All charged particles interacted via the full Coulomb potential
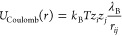
15where *r* is the distance between the charges *i* and *j*. The Bjerrum length is defined as
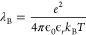
16where ϵ_0_ is the permittivity of vacuum. We set λ_B_ = 0.71 nm, which approximately corresponds to the relative permittivity of water ϵ_*r*_ = 78.5 at ambient temperature *T* = 298 K. The solvent was treated as a dielectric continuum, characterized by the relative permittivity *via* the Bjerrum length.

The electrostatic interactions were computed using the particle–particle particle-mesh (P3M) method.^58^ The algorithm was tuned to the relative accuracy of 10^–3^, using the automated tuning routine^59,60^ implemented in the software ESPResSo v4.1.4.^61^

### 2.2 Simulation Method

#### 2.2.1 Acid–Base Reactions

The acid dissociation reaction, eq 9, was modeled using the reaction ensemble method.^62^ In the forward direction, the trial Monte Carlo move entails changing the identity of the acidic group from HA to A^–^ and inserting an H^+^ ion into the system. In the reverse direction, the trial move entails changing the identity of the acidic group from A^–^ to HA and deleting a random H^+^ ion from the system. The trial move was accepted with a Metropolis-like probability^16,62^
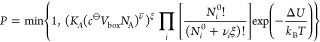
17where *K*_A_ = 10^–p*K*_A_^ is the acidity constant of the ionizable group, *c*^⊖^ is the reference concentration, *N*_*A*_ is the Avogadro number, *N*_*i*_ is the number of particles of type *i*, ν_*i*_ is their stoichiometric coefficient and ν̅ = ∑_*i*_ ν_*i*_. The symbol Δ*U* denotes the energy difference between the old and new configuration. The extent of reaction ξ = 1 corresponds to the deprotonation of the acidic sites (forward reaction in eq 9), and ξ = −1 corresponds to their protonation (reverse reaction in eq 9). To improve the sampling efficiency, alternative implementations of this reaction were employed simultaneously, as detailed in ref (16)

#### 2.2.2 Coupling to the Reservoir of Ions

The exchange of small ions between the system and the reservoir was modeled by inserting and deleting electroneutral ion pairs, formally described as virtual chemical reactions
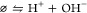
18
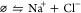
19
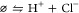
20
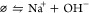
21where the empty set symbol, ⌀, denotes that the ions are taken from the virtual reservoir. In the forward direction of each of these reactions, the corresponding pair of ions is inserted at a random position in the system. In the reverse direction, the pair of ions is deleted. The trial move is accepted using the same acceptance criterion as the acid–base reactions, eq 17. The equilibrium constants of the above reactions are related to the chemical potentials in the reservoir, as explained in ref. (16). If the reservoir is a simple ionic solution, then these equilibrium constants are related to the concentrations of ions in the reservoir as
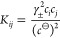
22where indices *i* and *j* refer to the involved ions and *c*^⊖^ = 1 mol/dm^3^ is the reference concentration. The mean activity coefficient is defined as , where γ_*i*_ is the activity coefficient of ion *i*. Specifically for eq 18, the equilibrium constant is fixed to
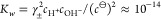
23Among the remaining three constants, two can be chosen independently to determine the pH and salt concentration in the reservoir. The last one follows from the electroneutrality constraint. To obtain the desired pH and salt concentrations, we used the empirical Davies equation to estimate the activity coefficients
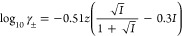
24We performed auxiliary simulation of the reservoir to check that in the range of simulated ionic strengths, the actual concentrations in the reservoir did not significantly differ from the desired ones.

#### 2.2.3 Simulation Protocol

All simulations were performed using the software ESPResSo v4.1.4.^61,63^ We simulated the systems using a combination of Langevin dynamics (LD) to sample the configurational space and the Grand-Reaction Monte Carlo (G-RxMC) method^16^ for simulating the acid–base equilibria and the exchange of ions between the retentate and the permeate as illustrated in Figure 1. For the Langevin dynamics integration, masses of all particles were arbitrarily set to 1 in the internal units of the simulation. This choice has no impact on the partition function and on the measured observables.

One simulation cycle consisted of 1000 LD integration steps with a time step *δt* = 0.01τ, where τ is the Lennard-Jones time unit, followed by 250 G-RxMC trial moves, including both acid ionization reaction and exchange of ions with the reservoir. The friction coefficient in the Langevin dynamics was set to Γ = 1/τ. For the nanoparticles, the Langevin thermalization was applied only to the core but not to individual ionizable sites. A typical simulation consisted of 5000 cycles, *i.e., t*_sim_ = 5 × 10^4^τ of Langevin dynamics time evolution and 1.25 × 10^6^ reaction trial moves. The configurations for postprocessing and computing observables were stored with time intervals *t*_obs_ = 20τ. We verified by visual inspection of the time evolution of various observables that all of them have equilibrated within less than about 10% of the total simulation time. These observables included total, kinetic, potential and electrostatic energy, the ionization degree of the ionizable sites, and the number of small ions. We noted that, at pH values far from the p*K*_A_ of the ionizable groups, the total energy and its components were typically the slowest evolving observables. Conversely, at pH values near the p*K*_A_, the ionization degree of the sites and the number of small ions were the slowest to equilibrate. Furthermore, we quantified the relaxation time (autocorrelation time) of each observable using the block analysis method, as described in ref (64). This procedure was also used to estimate the statistical uncertainty of the computed averages (statistical error of the mean). Eventually, we discarded the first 30% of the total simulation time as equilibration. This 30% threshold was chosen as a safe margin, much longer than the estimated equilibration times and relaxation times, yet not significantly affecting the estimated statistical error of the mean. The total simulation time was typically at least 100 times longer than the autocorrelation time of the ionization degree, which was also estimated using the block analysis. Ultimately, the above approach yielded statistical error estimates which were comparable to symbol size used in the figures.

## 3 Results and Discussion

We start the discussion by demonstrating how the distribution of small ions between retentate and permeate is determined by the concentration (volume fraction) of colloidal particles with discrete charges uniformly distributed on the surface. Specifically, in Section 3.1 we quantify to what extent the partitioning is determined by the Donnan equilibrium, as compared to the corrections for activity coefficients and crowding effects. These results serve as a reference for Section 3.2, where we discuss to what extent the Donnan effect and polyelectrolyte effect determine the charge on the nanoparticles with uniformly distributed ionizable groups at various pH values and ionic strengths. Finally, we show in Section 3.3, how the effects discussed in the preceding sections are affected by the patchy distribution of ionizable groups on the nanoparticle surface.

### 3.1 Permanent and Uniformly Distributed Charges

**Figure 2:**
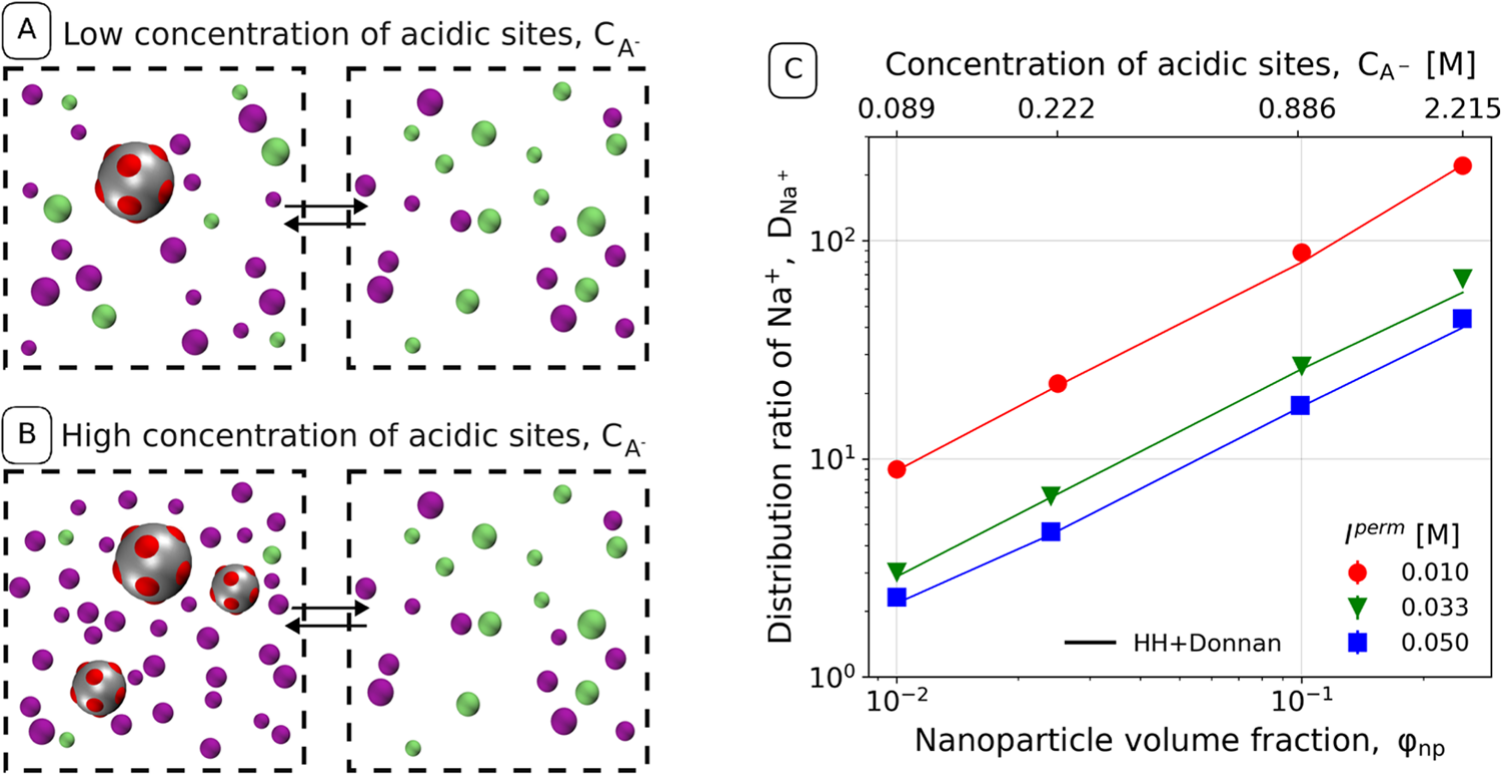
Schematic representation of the Donnan effect on the partitioning of small ions in systems with (A) low and (B) high concentration of acidic sites, *c*_A^–^_. Color code is the same as that in Figure 1. (C) Distribution ratio of Na^+^ between the retentate and the permeate, varying nanoparticle volume fraction (bottom axis) or its equivalent concentration of acidic sites (top axis) at various ionic strengths in the permeate. Different symbol shapes and colors encode ionic strength in the permeate. The solid lines represent the result of Donnan theory using the ideal gas approximation. The estimated statistical errors are comparable to the symbol size.

The situation at low concentration of nanoparticles with permanently charged acidic groups is schematically shown in Figure 2A. Under these conditions, only a small number of monovalent cations is required to compensate the nanoparticle charge. Consequently, their partitioning between the retentate and permeate is approximately equal. On the contrary, Figure 2B shows that a high nanoparticle concentration requires a high number of monovalent cations to compensate their charge, which leads to an strongly asymmetric distribution of small ions between both compartments. In the following, we provide a quantitative description of this effect.

Figure 2C shows the distribution ratio of sodium cations as a function of nanoparticle volume fraction, computed from the simulations (points). Different point shapes and colors correspond to different ionic strengths in the permeate. The double-logarithmic plot yields approximately straight lines, which is consistent with the expected trend from the Donnan theory *D*_+_ ≈ *c*_A^–^_^ret^/*I* in the concentration regime, where *c*_A^–^_ ≫ *I*. For reference, Figure 2C shows also analytical results from the ideal Donnan theory (solid lines), demonstrating that they almost quantitatively agree with the simulations. Therefore, we can conclude that in the range of nanoparticle volume fractions and ionic strengths studied here, the distribution ratios are very well predicted by the ideal theory, whereas the corrections for activity play a minor role. Nevertheless, they might play a more important role under different conditions, as has been discussed in the context of polyelectrolyte hydrogels^56^ and polyelectrolyte solutions.^16^

As follows from eq 5, the Donnan partitioning can be equivalently controlled by varying the concentration of nonexchangeable charges or by varying the ionic strength in the permeate. In accordance with that, we observe in Figure 2C high partition coefficients, *D*_+_ ≳ 10^2^ at high nanoparticle volume fractions, ϕ_np_ ≳ 0.1. In contrast, at low nanoparticle volume fractions, ϕ_np_ ≲ 10^–2^, the values of *D*_+_ approach unity. Due to symmetry considerations, identical results would be obtained for nanoparticles with basic (cationic) sites, albeit with the roles of cations and anions reversed.

At this point, it is worth highlighting that the key parameter determining the magnitude of the Donnan effect is the concentration of nonexchangeable charges, not the volume fraction of the nanoparticles. These two quantities are related by a constant proportionality factor if the net charge on the nanoparticles is fixed. Then, the variation of nanoparticle volume fraction is equivalent to variation of the concentration of nonexchangeable charges. However, the relation between them becomes nonlinear, if the charge on the nanoparticle depends on the pH. This topic will be addressed in the upcoming subsections.

**Figure 3:**
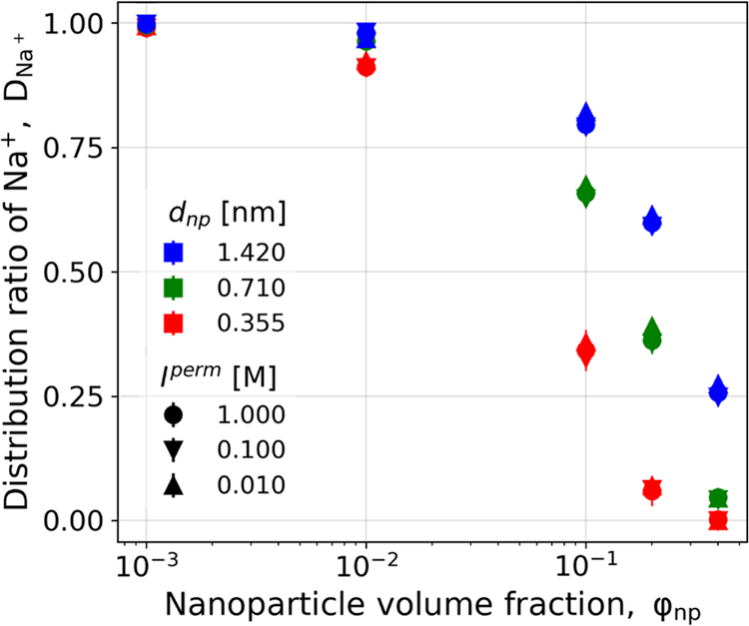
Distribution ratio of Na^+^ between the retentate and permeate at various volume fractions of the uncharged nanoparticles and various ionic strengths of the permeate. Different symbol shapes encode ionic strengths in the permeate, while different colors encode nanoparticle sizes, as indicated in the legend. The estimated statistical errors are comparable to the symbol size.

At higher ionic strengths in the permeate or higher nanoparticle volume fractions, steric effects caused by the finite size of ions might become important. To understand to what extent crowding effects are affecting the distribution of small ions in our simulations, we ran equivalent simulations with electrostatic interactions turned off, i.e., using neutral nanoparticles of various sizes and at various volume fractions, so that the asymmetric partitioning was determined exclusively by the steric effects. Figure 3 shows that the extent of steric effects depends not only on the nanoparticle volume fraction but also on the size ratio between the nanoparticles and small ions. If the ion size is comparable to or just slightly smaller than the size of the nanoparticles, *d*_ion_ ≲ *d*_np_ then the steric effects become important already at ϕ_np_ ≳ 0.1. However, if the ions are much smaller than the nanoparticles, *d*_ion_ ≪ *d*_np_, then the steric effects become relatively weak, yielding *D*_+_ ≳ 0.8 up to rather high nanoparticle volume fractions, ϕ_np_ ≲ 0.1. Only the limit *d*_ion_ ≪ *d*_np_ is relevant for nanoparticles or proteins in salt solutions. Therefore, we can conclude that the distribution of small ions in the solutions of charged nanoparticles is primarily controlled by the concentration of the nonexchangeable charges, whereas the steric effects play only a minor role.

Various theoretical models of protein dialysis invoked volume exclusion (crowding) effects as a possible source of discrepancy between theoretical predictions and experimental observations.^3,5−7,11^ For example, Briskot et al.^5^ suggested that neglecting the finite size of small ions might be an important source of these discrepancies, which could be augmented by combining the Poisson–Boltzmann description with a Stern layer in the immediate vicinity of the protein surface. In their study, they considered monoclonal antibodies, with the radius of 4.8 nm, which is more than 20 times bigger than small ions and also much bigger than nanoparticle radii considered in our Figure 3. By extrapolating our results in Figure 3 to an even bigger difference between the size of nanoparticles and small ions, it seems that volume exclusion effects due to the finite size of ions should be very small in solutions of monoclonal antibodies. Therefore, it is highly probable that the discrepancies between theory and experiment were caused by neglecting some other aspects than the finite size of small ions.

### 3.2 pH-Dependent and Uniformly Distributed Charges on the Nanoparticles

**Figure 4:**
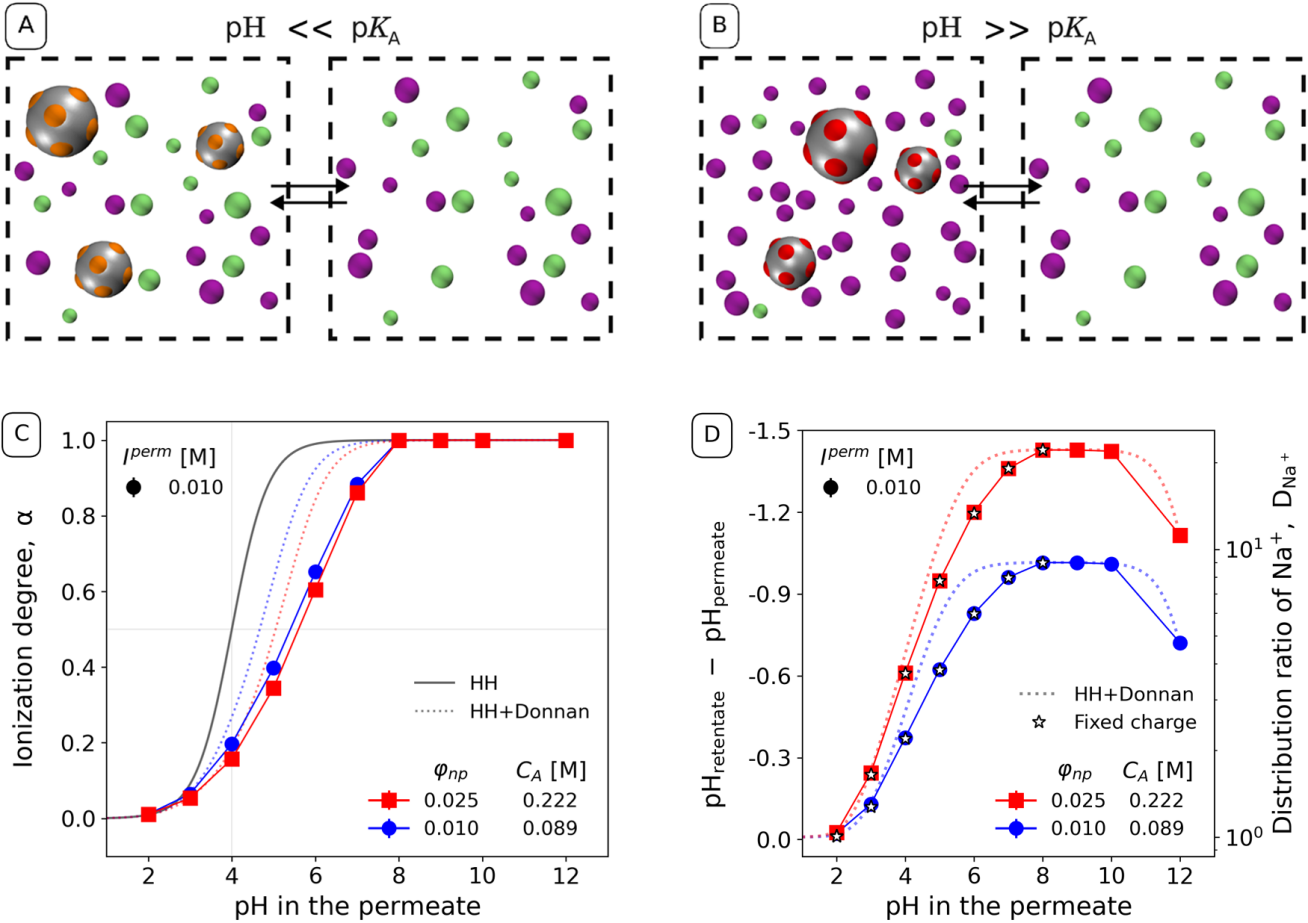
Schematic representation of the distribution of small ions at various pH values at (A) pH < p*K*_A_ and (B) pH > p*K*_A_. Color code is the same as Figure 1. (C) Ionization degree of the nanoparticles as a function of the pH in the permeate. (D) ΔpH (left axis) and the corresponding distribution ratio of Na^+^ (right axis) as a function of pH in the permeate at various nanoparticle volume fractions, ϕ_np_. Because ΔpH = −log_10_*D*_Na^+^_, both axes are linked to the same data, just expressed as different quantity. Different colors encode nanoparticle volume fraction (concentration of acidic sites), as indicated in the legend. The black solid line in the ionization degree plot corresponds to the ideal Henderson–Hasselbalch equation (eq 11). The dotted colored lines in both plots correspond to eq 11 corrected by ΔpH from eq 4. The star-shaped markers correspond to the simulation of systems with a fixed fractional charge, as described in the text. The estimated statistical errors are comparable to the symbol size.

Let us first consider nanoparticles with ionizable weakly acidic sites with p*K*_A_ = 4.0, uniformly distributed on the surface. At a fixed volume fraction of the nanoparticles, the concentration of nonexchangeable anionic charges is influenced by pH in the retentate via the acid–base reaction (eq 9). Simultaneously, according to eq 4, the difference between pH in the retentate and in the permeate is determined by the distribution ratio of monovalent cations (H^+^ or Na^+^). Figure 4A illustrates the situation at pH ≪ p*K*_A_, when the acidic sites are mostly nonionized, leading to an almost symmetric distribution of small ions between the retentate and permeate. Figure 4B illustrates the situation at pH ≪ p*K*_A_, when the acidic sites are mostly charged, causing a strongly asymmetric distribution of small ions. In addition to the Donnan equilibrium, which determines the value of ΔpH, this acid–base reaction is affected by electrostatic interactions between neighboring ionizable sites and by electrostatic screening due to the interaction with salt ions, resulting in a complex feedback loop.

Figure 4C shows that in a system containing weakly acidic groups, the ionization degree α increases as pH in the permeate is increased. Figure 4D shows that this increase in ionization leads to an increase in the pH difference between the retentate and permeate, which determines the magnitude of the *Donnan effect*. Therefore, pH in the retentate is lower than in the permeate and the increase in α is slower than predicted by the ideal Henderson–Hasselbalch equation without the Donnan term (HH, black solid line). Furthermore, the simulation results exhibit a slower increase in α than predicted by ideal acid–base equilibrium corrected by ΔpH due to Donnan partitioning (HH+Donnan, dashed lines). The difference between ideal HH+Donnan prediction and simulation results is caused by direct electrostatic interactions between ionized sites on the nanoparticle surface, termed *Polyelectrolyte effect*. In a real system, both Donnan and Polyelectrolyte effect contribute to the net result. However, the existing theories describing acid–base equilibria in dialysis, have considered only the Donnan effect, while neglecting the Polyelectrolyte effect.^5,8,9^

By comparing simulation results with the Donnan theory coupled to Henderson–Hasselbalch equation, we can quantify magnitude of the Polyelectrolyte effect neglected by the theory. Figure 4C compares ionization degrees from the ideal HH + Donnan calculations with simulations at two different nanoparticle volume fractions. At the higher volume fraction, HH + Donnan predicts a stronger Donnan effect and consequently a stronger shift in the ionization. However, the simulations suggest that this stronger Donnan effect is compensated by a weaker Polyelectrolyte effect, yielding almost identical dependences of α on the pH at both volume fractions. The systematic difference between the simulations and HH+Donnan analytical theory increases as the pH is increased, accompanied by a concomitant increase in α. Under the specific conditions studied here, the Donnan and Polyelectrolyte effects have comparable magnitudes. The relative importance of these two effects can be tuned by varying the concentration of ionizable groups in the retentate, or by varying the ionic strength of the permeate, following the same arguments as presented in ref (16). in the context of polyelectrolyte solutions.

Figure 4D compares ΔpH, obtained by solving the analytical HH + Donnan equations, with the simulation results obtained by using fluctuating charges and another set of simulations using the fixed fractional charges. Notably, ΔpH is coupled to *D*_Na^+^_ via eq 4. Any alteration in the ionization degree, seen in Figure 4C, directly impacts the distribution of small ions and the ΔpH as shown in Figure 4D. Therefore, at pH ≲ 8, both ΔpH and distribution ratios keep increasing as function of pH, following the trend in the ionization degree, until they reach a plateau between pH 8 and 10. This plateau is followed by a decrease of ΔpH at higher pH values, although the acidic sites remain fully ionized (α = 1). This behavior occurs because such high pH values can be obtained only if the concentration of OH^–^ becomes comparable of that of the salt. Then, the ionic strength in the permeate increases as the pH is increased, suppressing the Donnan effect although the salt concentration remains constant.^15,16,65^

Finally, we compare our simulation results in Figure 4D with analogous simulations using a fractional charge, fixed to the value given by the average charge obtained from the simulation with fluctuating charges. The ΔpH values obtained from both models are very similar, indicating that, under the studied conditions, our model does not exhibit significant charge fluctuations. However, significant charge correlations and fluctuations have been observed in previous simulations of oppositely charged polyelectrolytes with fluctuating charges^23−25^ and colloids containing both acidic and basic charge patches.^22^ Therefore, we expect that charge correlations and fluctuations will become important in the future in simulations of more realistic models of protein solutions, which include both acidic and basic residues.

### 3.3 pH-Dependent and Heterogeneously Distributed Charges on the Nanoparticles

**Figure 5:**
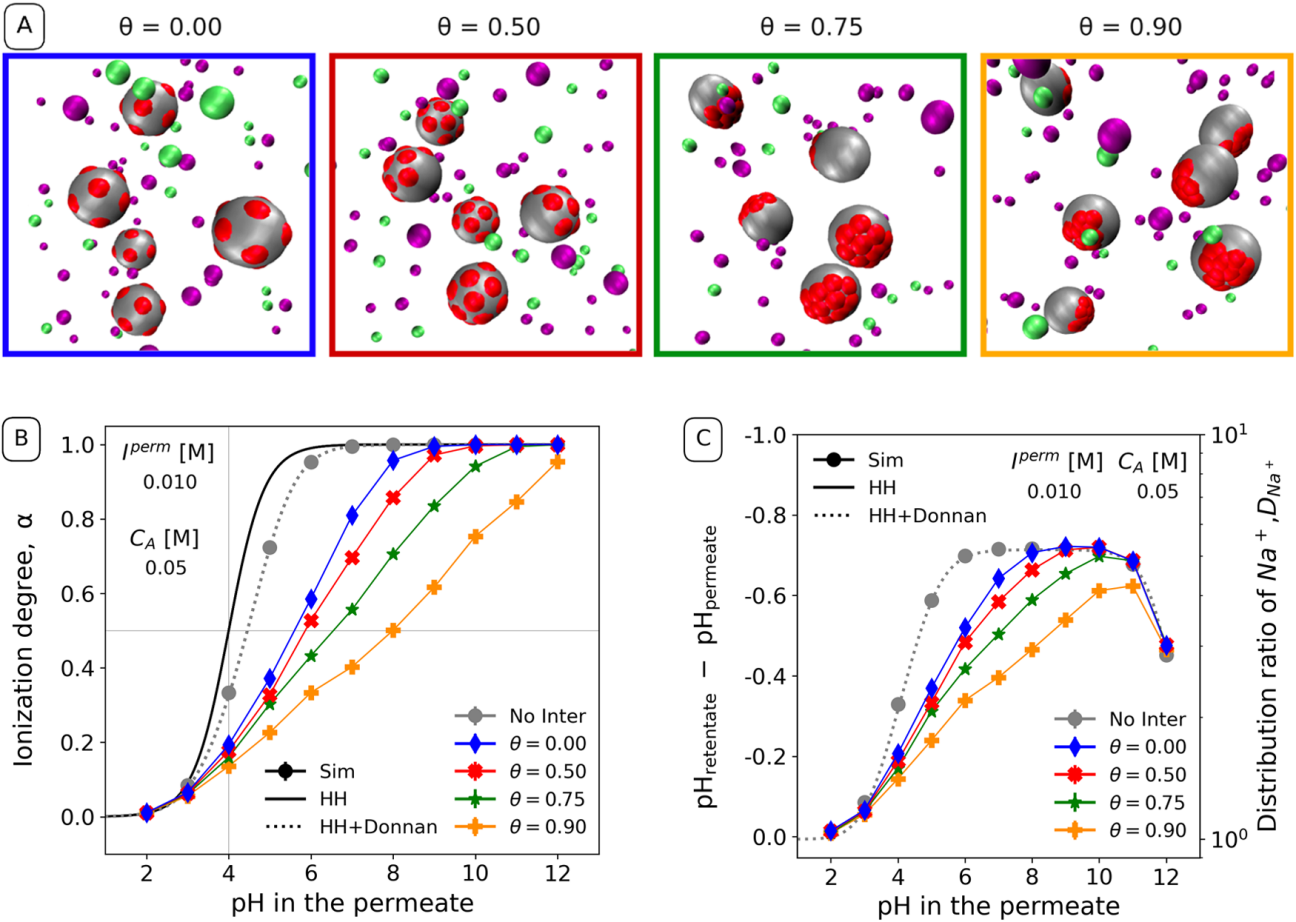
(A) Snapshots of the 4 different simulated charge distributions of the acidic ionizable groups on the nanoparticle surface. Color code is the same as that in Figure 1. (B) Ionization degree of the nanoparticles at different pH values. (C) Distribution ratio of Na^+^ between the retentate and permeate at various pH values. The gray data points correspond to the simulation results of a system with no interactions (No Inter). Different colors encode different degree of patchiness, θ, as indicated in the legend. The black solid line in the ionization degree plot corresponds to the ideal case computed by the Henderson–Hasselbalch equation (HH) and the dashed colored lines in both plots correspond to the ideal plus the Donnan contribution (HH+Donnan). The estimated statistical errors are comparable to the symbol size.

Finally, to understand the importance of the spatial distribution of charges on the nanoparticle surface, we decided to simulate systems with various degrees of charge patchiness, quantified by the patchiness parameter θ. The value of θ = 0 corresponds to charges uniformly distributed on the surface, yielding the same system as studied in the previous section. The simulation snapshots in Figure 5A illustrate changes in the distribution of ionizable sites as θ is varied from 0 through 0.50, 0.75, up to 0.90. The key parameter, which determines the effect of electrostatic interactions due to charge patchiness, is the distance between neighboring charges at a given value of θ, shown in Table 1. As a rule of thumb, if distances between charges are smaller or comparable to Bjerrum length, *d*_q_≪ λ_B_, then their electrostatic interaction energy is greater than *k*_B_*T* (cf. eq 15), which will strongly affect the acid–base equilibrium. On the contrary, at *d*_q_ ≫ λ_B_ the electrostatic interaction energy is smaller than *k*_B_*T* and its impact on the acid–base equilibrium should be rather small.

Figure 5B compares the ionization degrees of systems with the same volume fraction of nanoparticles, and thus the same concentration of nonexchangeable charges, but with different distributions on the nanoparticle surface. Under these conditions, the ideal HH+Donnan calculation produces always the same curve, irrespective of the charge distribution. However, the simulations account for site–site interactions, and therefore produce different curves for different values of θ. The results for the system with uniformly distributed acidic sites (θ = 0) exhibit the smallest deviation from the ideal behavior. This deviation progressively increases as the degree of patchiness θ increases. Notably, in the most patchy system simulated in our study (θ = 0.9) the shift extends up to 4 pH units at α = 0.5. As a result, patchy nanoparticles (θ > 0) require a higher pH to attain the same charge as nanoparticles with uniformly distributed ionizable groups (θ = 0).

In analogy with the previous subsection, we observe in Figure 5C that the lower degrees of ionization of the more patchy systems correspond to lower ΔpH and lower distribution ratios *D*_Na^+^_. Mirroring the trend in α(pH), the value of ΔpH increases slower and over broader range of pH when the patchiness is increased. Furthermore, Figure 5C shows that the plateau in ΔpH and *D*_Na^+^_ is not reached at θ ≳ 0.75. This is because the full ionization is not reached before the Donnan effect starts to decrease due to the increasing ionic strength.

## 4 Conclusions

In this study, we simulated a system containing charged colloidal nanoparticles in contact with a salt solution at a given pH. This model represents a solution of charged colloidal nanoparticles in a dialysis process. It can also serve as a highly simplified representation of a protein solution. The retentate, containing the colloids, is separated from the permeate by a semipermeable membrane which allows the exchange of small ions but prevents the exchange of the colloids. The charge on the colloids must be compensated by asymmetric distribution of small ions, termed the Donnan effect. We showed that up to high volume fractions of the colloids, ϕ_np_ ≈ 0.1, the Donnan effect dominates the distribution ratio of small ions, while steric (crowding) effects remain negligible. The crowding becomes important only at very high volume fractions of the colloids ϕ_np_ ≳ 10%, or if the colloids are small, so that their size is comparable to the monovalent ions. Interestingly, steric (crowding) effects have been invoked in various publications to explain the observed discrepancies between theoretical predictions and experiments.^3,5−7,11^ Because proteins are typically much smaller than monovalent salt ions, our results suggest that crowding effects are unlikely to be the primary cause of these discrepancies.

Subsequently, we focused on the effect of pH on the ionization degree of acidic sites on the colloids. We showed that the Donnan effect causes that pH in the retentate is lower than in the permeate, if the colloids are negatively charged. For symmetry reasons, the opposite would be true for positively charged colloids. In addition to the Donnan effect, the degree of ionization of the colloids is further decreased by the Polyelectrolyte effect, caused by electrostatic interactions between nearby ionizable sites on the same colloid. Depending on the colloid concentration and ionic strength of the permeate, both Donnan and Polyelectrolyte effect can be comparably significant or one of them may dominate. Current state-of-the-art theories of dialysis and ultra/diafiltration consider only the Donnan effect, while neglecting the Polyelectrolyte effect,^3,5,6,8^ which causes systematic deviations from the correct predictions. Therefore, we argue that the discrepancies between theoretical predictions and experimental observations in the dialysis of colloid and protein solutions should be attributed to the neglect of polyelectrolyte effect, rather than crowding.

Finally, we demonstrated how the Polyelectrolyte effect is related to distribution of ionizable sites on the nanoparticle surface. We showed that the importance of polyelectrolyte effect increases if the ionizable sites are located within a small patch, as compared to being uniformly distributed on the nanoparticle surface. This finding is important for further development of theories predicting the dialysis of proteins. Our results suggest that such a prediction can only be quantitatively correct, if it accounts for the inhomogeneous distribution of ionizable sites on the protein surface.

Our current model was designed as a proof of concept to demonstrate the importance of charge patchiness affecting the acid–base properties in protein solutions. Our nanoparticles were smaller than typical proteins and the charges were also closer than a typical distance between ionizable sites in proteins. Therefore, the effects observed here are stronger than what should be observed in protein solutions. Nevertheless, our results demonstrate that a similar type of model can be used to account for these effects in solutions of real proteins.

In order to use our approach for predicting the properties of real protein solutions, it is necessary to employ a more complex model than we used here. Such a model should use a distribution of charges on the nanoparticle which corresponds to a specific protein, matching not only the net charge but also its dipole, quadrupole or higher moments.^53,66^ Additionally, the employed p*K*_A_ values should reflect the actual residue composition of a protein, including both acidic and basic sites. Furthermore, it might be desired to include a more accurate representation of the shape of real proteins, such as the typical Y-shape of monoclonal antibodies, following the shape-based coarse-graining procedures.^67^ This can be achieved, e.g., using a set of interconnected spheres rather than a single sphere, better mimicking the geometry of real proteins. Moreover, the model of the dialysis buffer can be refined by including not only monovalent ions but also multivalent ions and accounting for their variable ionization states. This might be important for example in histidine-based buffers, which are often used in ultra- and diafiltration.^5^ Finally, it will be necessary to consider higher protein concentrations than investigated in the current study. Under these conditions, protein–protein interactions and crowding effects are likely to become important. However, our approach can be easily adapted to include all these effects, as soon as the structure of the protein is known, and the buffer composition is specified. Therefore, in combination with a realistic model of a protein, it should be able to quantitatively predict the dialysis of concentrated protein solutions also under conditions where the currently used models fail. In this respect, the approach presented here paves the way toward quantitative predictions of not only dialysis but also ultra- and diafiltration of concentrated protein solutions under various buffer conditions.

## Data Availability

The data supporting this article has been uploaded to a public repository: https://zenodo.org/records/14045608
